# Personal

**Published:** 1909-06

**Authors:** 


### PERSONAL.

---

Dr. Lewis S. McMurtry, of Louisville, by invitation, delivered an address before the American Gynecological Society at its recent annual meeting at New York, commemorative of the work of Ephraim McDowell, and especially of his first ovariotomy,—the first in the world,—performed at Danville, Ky., December, 1809. Dr. McMurtry lived many years at Danville, was chairman of the McDowell Monument Committee, and is better prepared than any other man living to perform the function imposed upon him.

Commenting upon the event, the New York Medical Journal in its issue of May 1, 1909, said:

The special event in the commemoration last week was the banquet, held on Thursday evening, and it was a happy thought to arrange that Dr. McMurtry's address should be given after the dinner, at a time when it naturally led to sympathetic remarks by other distinguished men. The choice of Dr. Lewis S. McMurtry, the eminent Louisville gynecologist, to speak in commemoration of the pioneer ovariotomist, also a Kentuckian, was peculiarly appropriate. Nobody could utter more telling or more graceful sentences concerning McDowell than those that came from Dr. McMurtry.

---

Dr. Vertner Kenerson, who has been absent from professional work since April, 1908, resumed his practice at No. 181 Allen

Street, Buffalo, May 1, 1909. His office hours hereafter will be from 1 to 4 P. M.; no morning or evening hours, except by appointment. Doctor Kenerson's private cottage hospital, open to the profession, for selected cases, is at No. 115 Park Street, Buffalo. Telephones at No. 181 Allen St., are Bell, Tupper 70, Frontier 70. At the Cottage, Frontier 13083.

---

Dr. P. W. Van Peyma, of Buffalo, has removed from 445 William Street to 242 Norwood Avenue. Telephones: Bell, Bryant 101; Frontier, 12492.

---

Dr. James Stoddart, of Buffalo, has removed his office from Ellicott Square to his residence, 470 Franklin Street.

---

Dr. and Mrs. Edward T. Smith, formerly of Fourteenth Street, Buffalo, have moved to their new home, No. 503 Porter Avenue.

---

Drs. Park and McGuire, whose offices are removed to 430 Delaware Avenue, as announced in the Journal for May, have installed a complete clinical laboratory, which is under the direction of Dr. B. T. Simpson. Adequate apparatus is also at hand for *x*-ray, finsen, radium, and fulguration therapy in suitable cases. Hours: 2 to 4 P. M. Sunday by appointment only.

Telephones: Bell, Tupper 4; Frontier (Federal), 637.

Dr. Park's residence, 510 Delaware Avenue. Telephones: Bell, Tupper 208; Frontier 3599.

